# Education Research: Observational Study of Educational Debt Among US Medical Graduates Entering Neurology, 2010–2021

**DOI:** 10.1212/NE9.0000000000200118

**Published:** 2024-03-13

**Authors:** Shubhdeep V. Ahden, Wasim Zatar, David N. Herrmann, E. Ray Dorsey, Benjamin P. George

**Affiliations:** From the University of Rochester Medical Center, NY.

## Abstract

**Background and Objectives:**

To identify trends in educational debt for US medical school graduates entering neurology and compare debt to anticipated compensation.

**Methods:**

Data of 148 accredited medical schools were obtained from the Association of American Medical Colleges Graduation and Matriculating Student Questionnaires to identify self-reported educational debt for graduates pursuing neurology training. Trends were assessed in a 2-year interval from 2010 to 2021. Descriptive statistics were used to assess characteristics associated with debt. Dollar amounts were adjusted to 2021 US dollars. Total compensation by subspecialty from the 2021 American Academy of Neurology Compensation and Productivity Survey was used to calculate debt-to-income ratios by subspecialty.

**Results:**

There were 182,738 responses recorded from medical graduates from 2010 to 2021, of which 4,466 planned on neurology training. The percentage of medical graduates entering neurology with debt decreased from 82% in 2010–2011 to 71% in 2020–2021. Among indebted, the median educational debt increased 9% from $192,613 (interquartile range [IQR] $140,908) in 2010–2011 to $209,396 (IQR $159,128) in 2020–2021 (*p* < 0.001). Medical graduates with debt more often reported family income in the bottom 3 quintiles of US household income (18% with debt vs 7% without debt; *p* < 0.001). Graduates from self-identified race and ethnicity groups who are underrepresented in medicine (URiM) were more likely to have debt (15% vs 9%; *p* < 0.001) and had greater debt when compared with graduates not self-identifying as URiM (median $211,616 [IQR $152,760] vs $202,379 [IQR $153,340]; *p* < 0.001). In 2021, 46% of indebted neurology-bound graduates indicated plans to use Public Service Loan Forgiveness, which increased from 16% in 2010. In 2021, the median debt for neurology graduates represented between 70% and 93% of total annual compensation with the highest debt-to-income ratios among behavioral neurology (0.93), child neurology (0.91), and movement disorders (0.89).

**Discussion:**

The burden of educational debt for neurology-bound graduates is increasingly concentrated among those from lower-income families and racial and ethnic groups who are URiM. Subspecialties that often manage patients in the outpatient setting, as compared with those that are primarily inpatient/procedural, may have greater debt with respect to their compensation. Nearly half of 2021 graduates pursuing neurology plan to use tax-payer funds for loan forgiveness.

## Introduction

Medical education debt rises annually and increases the debt burden on individual medical graduates,^1^ while physician payments have recently faced reductions.^2^ There is a lack of information on how educational debt for those entering neurologic specialties compares with physician income. It is especially important to understand educational debt for certain groups, including those who identify as underrepresented in medicine (URiM) who may be subject to greater debt levels^3^ and those who primarily care for outpatients with earnings based on low paying evaluation and management (E/M) services.^4,5^ More information on educational debt and income for those entering neurology is necessary to shape policies that will improve equity in the workforce and support E/M physician work.

Students enter a transaction with medical schools, in which the student or graduate pays for an education that evolves into skills and credentials which are subsequently sold to patients and society as health care services.^6^ Historically, rising medical education costs coincided with growth in physician payment.^7^ However, as physician payments face cutbacks,^2^ the medical education market teeters on an economic “bubble”—where the cost of medical education exceeds the value for its graduates.^6^ Little is known about medical education debt in the context of future earnings (i.e., debt-to-income) for medical graduates more recently or those entering neurology.^6,7^

In addition to the burden of time and financial investment in training faced by all physicians, individuals who identify with URiM races or ethnicities have been excluded from building wealth.^3^ Previous studies have shown that Black individuals have lower household income and lower accumulated wealth compared with White individuals.^8,9^ Therefore, individuals who self-identify with URiM groups may face greater debt burdens in their pursuit of medical education.^3^ These financial barriers are another obstacle to creating a more balanced workforce needed to care for an increasingly diverse population.^10^ Understanding disparities in medical debt for neurology-bound medical school graduates is imperative when training, recruiting, and fostering the careers of the next generation of neurologists.^11,12^

As medical education debt grows, new federal loan forgiveness programs have emerged.^13^ The Public Service Loan Forgiveness (PSLF) program is offered through the US Department of Education which promises to relieve the remaining loan forgiveness balance, tax-free, after completion of 120 qualifying loan payments (consecutive or nonconsecutive) while working for a 501(c)3 nonprofit institution (such as an academic or nonprofit community hospital), including payments while employed as a resident or fellow.^14,15^ However, significant political uncertainty surrounds this program, and as a recent policy addition, the program has yet to demonstrate effectiveness in large-scale loan forgiveness.^16^

This paper will focus on the educational debt faced by medical school graduates planning to enter training in neurology. Using a large census of US medical school graduates, we aim to (1) investigate trends in educational debt for neurology graduates over time, (2) examine groups disproportionately affected by debt, and (3) calculate debt-to-income ratios for various neurologic subspecialties. We hypothesized that the burden of educational debt is increasing with time, that historically underrepresented groups have greater debt burdens, and that nonprocedural subspecialties shoulder large debt amounts compared with their incomes.

## Methods

We performed a retrospective cross-sectional observational study of educational debt among US medical graduates responding to the Association of American Medical Colleges (AAMC) Matriculating Student Questionnaire (MSQ) and Graduation Questionnaire (GQ) from 2010 to 2021.^17,18^

### Standard Protocol Approvals, Registrations, and Patient Consents

This study was considered exempt from federal regulation for protecting human research participants by the University of Rochester Research Subjects Review Board. Since this study was an analysis of previously collected deidentified data, consent was not obtained.

### Data Source for Educational Debt

The AAMC GQ is an annual cross-sectional census of all graduating medical students at US medical schools accredited by the Liaison Committee on Medical Education (LCME). The GQ contains information on educational debt, scholarship, and the respective dollar amounts. The AAMC MSQ is an annual cross-sectional census of all matriculating students at US medical schools accredited by the LCME. The GQ was linked to the corresponding MSQ responses for each graduating medical student to obtain medical student-reported family income. We examined deidentified data from the GQ and MSQ as completed by over 180,000 graduating medical students from 2010 to 2021. The overall average response rate from 2010 to 2021 was 71.1% for the MSQ and 81.1% for the GQ. The specific questions from the questionnaires used for the study are outlined in eAppendix 1 (links.lww.com/NE9/A60).

### Data Source for Subspecialty Compensation

To compare educational debt to physician income for neurologic subspecialties, we obtained data on total (pretax) compensation from the American Academy of Neurology Compensation and Productivity Survey for 2021.^19^

### Graduate Characteristics

Age, sex, race and ethnicity, degree program (e.g., MD, MD/PhD), public or private school ownership, and receipt of scholarship were available directly from the GQ and self-reported by the graduate. Although the AAMC now collects information on gender diversity from medical students in its most recent edition of surveys, the AAMC GQ and MSQ from 2010 to 2021 is limited to self-reported “sex assigned at birth,” and therefore, we were unable to identify gender diversity. We defined underserved practice plans as an affirmative response to practice in underserved locations or to practice for an underserved population as obtained from the GQ and self-reported by the graduate. Consistent with prior studies, we defined URiM as Black, Hispanic, Native American or Alaskan Native, or Native Hawaiian or other Pacific Islander.^10,12^ Owing to the risk of deidentifying graduates, specific race and ethnicity could not be revealed due to small numbers within categories. Information on family income was available in the MSQ for 64% of GQ respondents. We classified family income into quintiles based on the year of the MSQ matched to quintiles of US household income as reported by the US Census Bureau.^20^ The GQ also contained information on the possession of home mortgages for respondents graduating from 2015 to 2021.

### Outcomes

We examined the presence and amount of educational debt, which was calculated as the summation of premedical and medical education debt, as reported by graduates within the GQ. We also examined debt-to-income ratios, which we defined as total educational debt on graduation as a proportion of total annual compensation by neurology subspecialty assuming the median debt amount for graduates entering neurology in 2021. This definition is consistent with debt-to-income ratio calculations used for education debt in prior literature.^6,7^ Finally, among indebted graduates, we examined the percentage indicating intent to use the US Department of Education's PSLF program.

### Medical Graduate Selection

**Figure 1:**
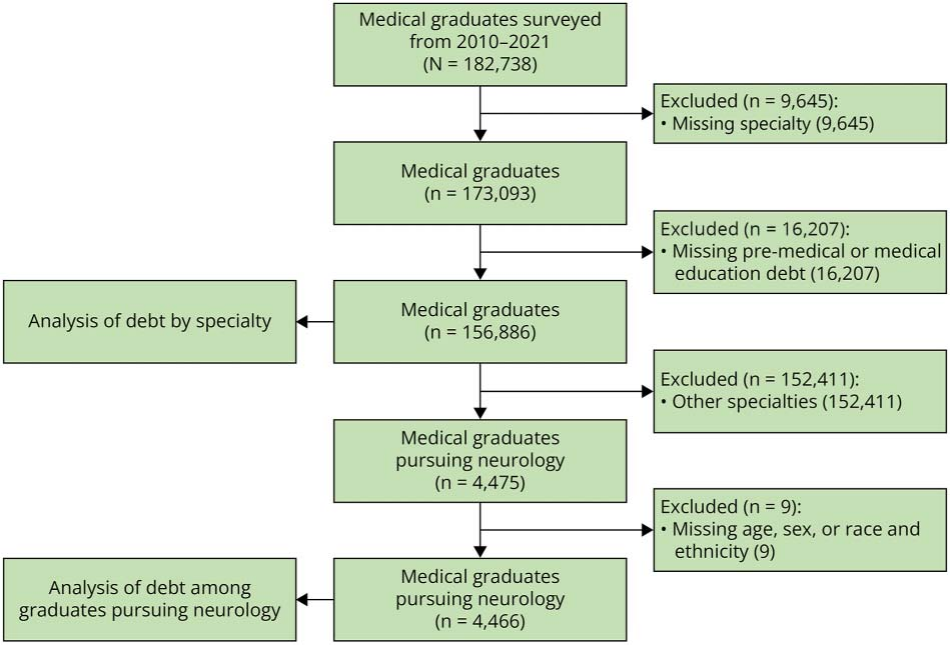
US Medical Graduate Selection Methods For an analysis comparing educational debt among graduates entering Neurology to those entering other specialties, we examined responses from 156,886 US medical graduates. For an analysis of trends of medical education debt and characteristics associated with debt, we examined responses from 4,466 US medical graduates indicating Neurology as their chosen specialty.

For an analysis comparing educational debt among neurology graduates to those entering other specialties, we examined responses in the GQ from 156,886 US medical graduates with known educational debt. To analyze trends of medical education debt and characteristics associated with debt, we examined responses from 4,466 US medical graduates indicating neurology as their chosen specialty (see flowchart in Figure 1).

### Statistical Analysis

Dollar amounts were adjusted to 2021 US dollars using the US Bureau of Labor Statistics Consumer Price Index for All Urban Consumers.^21^ Differences in categorical variables were assessed using χ^2^ tests. Differences in continuous variables were assessed using Kruskal-Wallis tests. Trends were assessed in a 2-year interval using a Cochran-Armitage test of trends. Analyses were conducted using Stata/MP version 17.0 (StataCorp, College Station, TX). Statistical tests were 2-sided with significance set, a priori, *p* < 0.05.

### Data Availability

Data availability on request to the authors is restricted by a data licensing agreement with the AAMC. Data are available to the public for request and purchase for a nominal processing fee through AAMC website.^22^

## Results

### Educational Debt in Neurology Compared With Other Specialties

**Figure 2:**
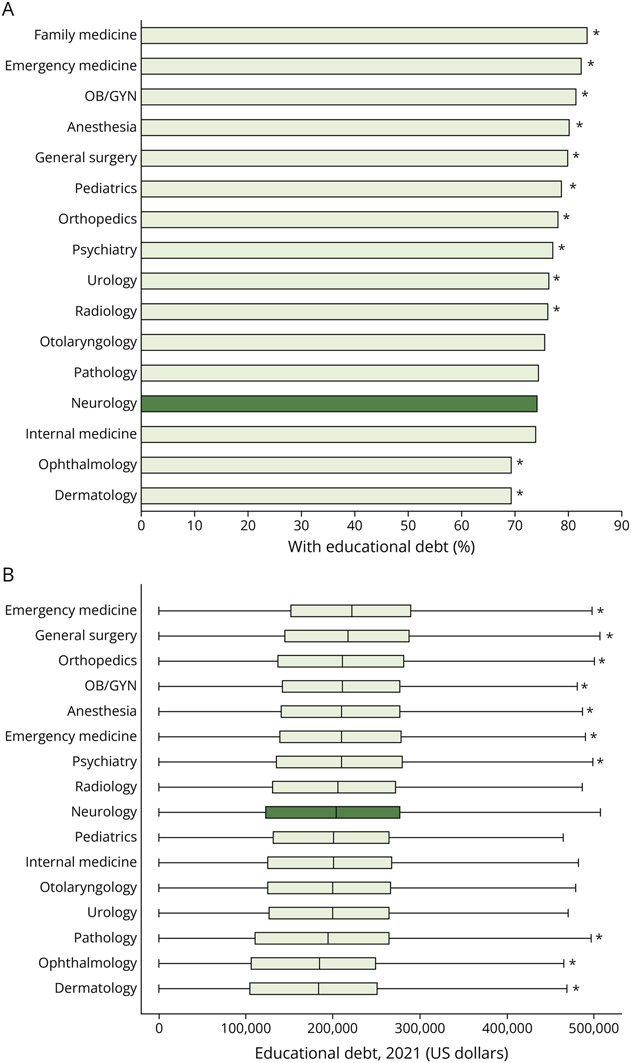
Educational Debt for US Medical Graduates by Specialty (A) Proportion of medical graduates with educational debt. (B) Boxplot of educational debt among those with debt adjusted to 2021 US dollars. Box represents median and interquartile range. Educational debt includes both premedical and medical educational debt. Figure represents n = 146,901 medical graduates classified into specialties based on postgraduate plans. There were n = 9,985 graduates who were not classified, reported “other” specialties, or did not intend to pursue medicine. Neurology is indicated in dark green. All specialties were compared with Neurology with asterisk indicating differences, *p* < 0.05.

There were 156,886 US medical graduates selected from the AAMC databases from 2010 to 2021. Graduates planning to enter neurology were among those with the smallest percentage with educational debt at 74%, ranging from 69% for dermatology and ophthalmology and 84% for Family Medicine (Figure 2A). The median debt balance for indebted graduates entering neurology was $203,527 (IQR $121,600–$276,365). From 2010 to 2021, 18% of indebted neurology-bound graduates had debt over $300,000. Among indebted graduates, those planning to enter emergency medicine had the greatest debt balances and those planning to enter dermatology had the smallest debt balances, with neurology near the middle (Figure 2B). Total educational debt incurred by this cohort of neurology-bound medical graduates increased from $42.9 million in 2010 to $69.7 million in 2021, representing 2.3% and 3.1% of all medical education debt in 2010 and 2021, respectively.

### Characteristics of Neurology Graduates by Educational Debt

There were 4,466 medical graduates in the cohort planning to enter neurology residency who filled out a graduation questionnaire at the time of their graduation from 2010 to 2021. The Table presents graduation characteristics stratified by the presence or absence of educational debt at the time of medical school graduation and educational debt balances among those indebted at the time of graduation. There were 26% (n = 1,159) of neurology graduates leaving medical school with no educational debt from 2010 to 2021. Characteristics associated with having educational debt (compared with having no debt) included self-identifying with URiM races or ethnicities, lower quintiles for family income, attending publicly owned medical school, planning to practice in underserved areas or work for underserved populations, having received any scholarship during medical school, and having a home mortgage. Characteristics associated with greater debt balances among those indebted were similar with few exceptions. Factors associated with greater debt balances among indebted included male (compared with female), private school graduates (compared with public school graduates), and those reporting no scholarship (compared with receiving any scholarship) (Table).

**Table:** Educational Debt of US Medical Graduates Entering Neurology by Graduate Characteristics

Characteristics	Educational debt (n = 3,307), n (%)	No educational debt (n = 1,159), n (%)	*p* Value^a^	Debt in US$ among indebted,^b^ median (IQR)	*p* Value^a^
Age, y					
≤25	415 (13)	183 (16)	<0.001	191,924 (126,482)	<0.001
26–27	1,535 (46)	480 (41)		201,792 (140,283)	
28–29	707 (21)	232 (20)		217,218 (150,113)	
≥30	650 (20)	264 (23)		201,508 (208,542)	
Sex assigned at birth^c^					
Female	1,766 (53)	621 (54)	0.916	197,891 (153,340)	<0.001
Male	1,541 (47)	538 (46)		211,979 (152,760)	
Underrepresented in medicine^d^					
Yes	502 (15)	105 (9)	<0.001	211,616 (156,901)	0.009
No	2,805 (85)	1,054 (91)		202,379 (155,898)	
Family income, quintile^e^					
First or second quintile (0%–40%)	304 (9)	29 (3)	<0.001	216,245 (173,823)	<0.001
Third quintile (41%–60%)	308 (9)	52 (4)		225,401 (136,828)	
Fourth quintile (61%–80%)	554 (17)	101 (9)		200,153 (152,816)	
Fifth quintile (81%–95%)	590 (18)	170 (15)		213,947 (149,177)	
Fifth quintile (96%–100%)	440 (13)	319 (13)		183,137 (155,768)	
Not reported	1,111 (34)	488 (42)		200,000 (155,953)	
Degree program					
MD	2,974 (90)	859 (74)	<0.001	209,476 (145,089)	<0.001
MD/PhD	190 (6)	240 (21)		39,883 (92,148)	
MD, postbaccalaureate	64 (2)	39 (3)		200,035 (144,618)	
MD with masters	79 (2)	21 (2)		230,336 (163,340)	
School ownership					
Private	1,392 (42)	555 (48)	0.001	215,820 (172,630)	<0.001
Public	1,915 (58)	604 (52)		197,740 (143,191)	
Underserved practice plans					
Yes	970 (29)	220 (19)	<0.001	213,792 (156,976)	<0.001
No	1,121 (34)	457 (39)		190,781 (156,237)	
Unknown	1,216 (37)	482 (42)		205,029 (144,888)	
Scholarship					
Yes	2,254 (68)	573 (49)	<0.001	198,983 (151,979)	<0.001
No	1,053 (32)	586 (51)		214,512 (153,497)	
Home mortgage (2015–2021)					
Yes	534 (16)	109 (9)	<0.001	233,177 (175,012)	<0.001
No	1,625 (49)	788 (68)		203,222 (145,000)	

Abbreviations: AAMC = Association of American Medical Colleges; GQ = Graduation Questionnaire; IQR = interquartile range; MSQ = Matriculating Student Questionnaire.

a Comparisons with and without educational debt across groups were made by the χ^2^ test. Comparisons for educational debt amounts across groups were made using the Kruskal-Wallis test.

b Educational debt values are reported in 2021 US dollars. Individuals without education were excluded from the calculation of median/IQR debt values.

c Gender identity is not available in the AAMC GQ or MSQ from 2010 to 2021.

d Underrepresented in Medicine category includes individuals who self-identified as Black, Hispanic, Native American or Alaskan Native, or Native Hawaiian or other Pacific Islander. Individual races are not displayed due to low n-values and concern for loss in data confidentiality.

e Family income quintiles are classified based on parental income reported in the AAMC matriculating student questionnaire matched by year to quintiles of US household income from the US Census Bureau. First and second quintiles are consolidated due to low n-values in the categories and concern for loss in data confidentiality.

### Trends in Educational Debt Among Neurology Graduates

**Figure 3:**
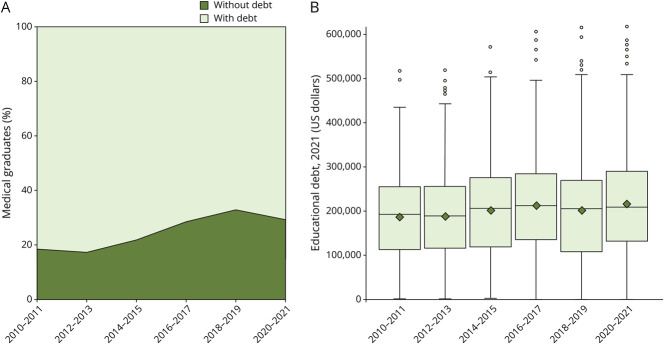
Trends in Educational Debt Among US Medical Graduates Entering Neurology, 2010–2021 (A) Percent with and without educational debt. (B) Box and whisker plot of educational debt among those with debt adjusted to 2021 US dollars. Mean values shown as diamonds. Trends were consolidated into a 2-year interval due to size of the categories. A Cochran-Armitage test of trends demonstrated increasing debt over time, *p* < 0.001.

The percentage of medical graduates entering neurology with debt decreased from 82% in 2010–2011 to 71% in 2020–2021 (*p* < 0.001) (Figure 3A). Among those with debt, the median educational debt increased from $192,613 (IQR $140,908) in 2010–2011 to $209,396 (IQR $159,128) in 2020–2021 (*p* < 0.001) (Figure 3B). Greater skew in the distribution of debt over time has led to a 16% increase in the mean debt among indebted, from $187,987 (SD 100,807) in 2010–2011 to $217,159 (SD 117,612) in 2020–2021 (Figure 3B).

### Debt-to-Income Ratios by Neurologic Subspecialty and Practice Setting

**Figure 4:**
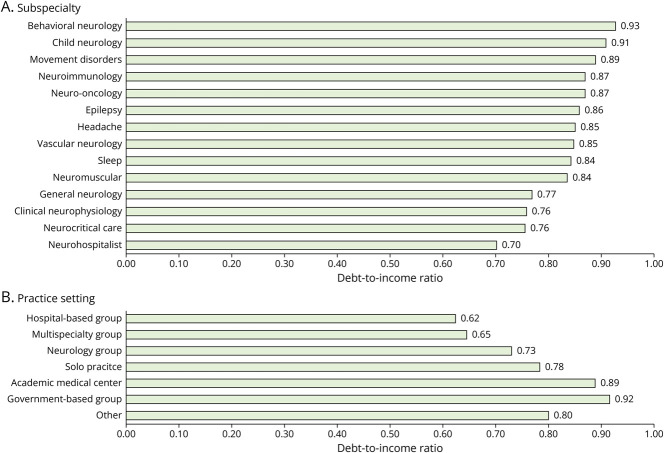
Ratio of Debt-to-Income by Neurologic Subspecialty and Practice Setting, 2021 We calculated debt as a proportion of total annual compensation by neurologic subspecialty and practice setting assuming the median debt amount for US medical graduates entering Neurology in 2021 and total compensation from the American Academy of Neurology Compensation and Productivity Survey. Subspecialties with fewer than 50 responses were excluded (Neuro-Ophthalmology, Pain Medicine, Endovascular Neurology, Autonomic Disorders, Geriatric Neurology, Other).

The median annual total compensation for neurologic subspecialties ranged from $216,000 for behavioral neurology to $285,000 for neurohospitalists in 2021. Debt-to-income ratios by neurologic subspecialties are shown in Figure 4A. The median educational debt ranged from 70% to 93% of the median total pretax annual compensation for neurologic subspecialties in 2021. The largest debt-to-income ratios were among movement disorders (0.89), child neurology (0.91), and behavioral neurology (0.93). The smallest debt-to-income ratios included neurohospitalists (0.70), neurocritical care (0.76), and clinical neurophysiology (0.70).

The median annual total compensation by practice setting ranged from $218,000 for government-based groups to $320,000 for hospital-based groups in 2021. Debt-to-income ratios by practice setting are shown in Figure 4B. The largest debt-to-income ratios were among those practicing in government-based groups (0.92) and academic medical centers (0.89). The smallest debt-to-income ratios were noted among hospital-based groups (0.62) and multispecialty groups (0.65).

### Use of PSLF by Neurology Graduates

The percentage of indebted graduates indicating intent to use PSLF nearly tripled from 16% in 2010 to 46% in 2021.

## Discussion

The results of our study show an increasing proportion of medical graduates entering neurology without debt, from 18% in 2010–2011 to 29% in 2020–2021. Graduates with debt were more likely from families with lower parental income. Conversely, graduates without debt were more likely from families with higher incomes. These findings suggest medical graduates entering neurology are increasingly from wealthier backgrounds, similar to findings of previous studies for all specialties.^23^ Over the past decade, approximately half of medical school enrollees come from families in the top quintile of US household income and less than 5% are from families in the bottom quintile for income.^24^ Careful attention is needed to the trends in the economic diversity of US medical graduates to promote a more balanced, equitable, and representative physician workforce.

Despite more graduates completing medical school without debt, we found debt balances per capita are increasing among the indebted.^25^ Accounting for inflation, the mean educational debt among indebted neurology graduates increased by $29,000 from 2010 to 2021, and nearly 1 in 5 graduates have debt in excess of $300,000. In the face of increasing medical education costs, these findings indicate that educational debt is increasingly concentrated among a smaller group of medical graduates who cannot afford to pay the rising “sticker price” of medical school without educational loans. This indicates that the emotional, psychological, and financial burden of debt on individuals will disproportionately affect some doctors more than others.^26^

Among graduates who identified with URiM groups, 82% reported educational debt, compared with 73% among other groups. Graduates identifying with URiM groups reported median debt balances of $212,000, compared with $202,000 for other groups. Inequities in educational debt serve as a barrier to accessing medical education for those that may have been historically excluded from generating wealth.^3^ As debt balances grow among fewer individuals, this concentration of the debt burden may widen inequities and counter efforts to improve workforce diversity.^10^ Many residency programs, departments, and institutions have created initiatives to improve diversity, equity, and inclusion.^11,27^ While this may lead to increased recruitment of URiM graduates, more work is needed to bridge the socioeconomic gap. Hiring practices could be expanded to offer scholarships, reduced tuition, or loan forgiveness options for URiM candidates. Policymakers should focus on the development or enrichment of programs designed to improve access to scholarships or loan repayment for URiM groups.^28^

We examined median cumulative US medical graduate educational debt for those entering neurology on graduation with respect to median annual pretax physician compensation by subspecialty. We refer to this metric as a debt-to-income ratio. Previous work demonstrated that debt-to-income ratios were approximately 0.5 for medical specialties and 0.7 for primary care specialties in 2010.^7^ Our study finds that in 2021, debt-to-income ratios range from 0.7 to 0.9 for neurologic subspecialties and are highest among those who commonly care for patients in the outpatient setting (e.g., behavioral neurology, movement disorders) with limited opportunities for procedural reimbursements and those within government-based practices and academic medical centers. The implications of these findings include the potential for educational debt to influence subspecialty choice,^29^ which may not reflect societal needs. Our current system rewards physicians providing acute care services,^4^ although small changes in the physician payment structure are beginning to address these issues by increasing the value of E/M services.^5^ However, if prospects for physician income fall rapidly or do not keep pace with medical education costs, then schools will need to re-evaluate the processes and costs of producing a physician.^6^

This is further complicated by “masking” of medical education costs with broad-sweeping loan forgiveness programs.^15^ For example, the Department of Education's PSLF program has allowed students to be comfortable acquiring greater debt balances with the promise of forgiveness after 120 income-based monthly payments while working for a 501(c)3 tax-exempt organization (e.g., including payments while working in an academic hospital tied to a medical school where debt was amassed).^30^ In addition to unlimited forgiveness amounts offered by PSLF, there are several federal, state, and hospital/clinic employer-level programs that provide limited loan forgiveness options with variable restrictions or contingencies.^13^ These programs, such as PSLF, may offer solutions for some but introduce biases and inequities (e.g., greater forgiveness amount for highly paid surgeons with long training periods) while blinding us to rising medical education costs.^15^ The political uneasiness surrounding the PSLF program is palpable, leaving a trail of uncertainty for borrowers intending to use the program.^16,31^

Nonetheless, 1 in 2 medical graduates with educational debt entering Neurology residency in 2021 plan to use PSLF, as opportunities for employer-specific loan repayment options in either the academic or private workforce may be scarce and insufficient to cover large debt balances.^13^ However, there have been some changes that have the potential of leaving significant impact on those knowing about and qualifying for PSLF. The Temporary Expanded PSLF allows for forgiveness for those who were either previously rejected or may not have qualified for PSLF due to previously nonqualifying payment plans.^32^ The Limited PSLF Waiver and the Income-Driven Repayment Account Adjustment also expanded PSLF temporarily by allowing for consolidation of non-Direct student loans with Direct student loans, counting payments within certain forbearance categories, allowing borrowers to “buy back” previously nonqualifying deferment periods, expanding criteria for qualifying employment, and relaxing rules for qualifying payments (i.e., late payments or installments).^33^ Furthermore, the US Department of Education has implemented multiple changes to loan servicer regulations and contracts to improve borrower experiences.^34^ The political trend in the past 4 years has been an overall expansion of federal loan forgiveness.

This study has several important limitations. Our data are limited to graduates of allopathic medical schools and are not generalizable to the entire US neurology workforce. Osteopathic schools may account for a large and growing percentage of graduates entering neurology,^35^ and these individuals may be subject to even greater debt burdens due to the private ownership or affiliation of most osteopathic schools. Therefore, our study likely underestimates the overall burden of educational debt for neurology-bound medical school graduates. Graduate intent on specialization is collected at the time of survey completion which occurs during the fourth year of medical school either shortly before or after matching into a training program. We cannot verify the transition from medical school to residency using these data; however, given the timing of the survey, we estimate that close to 100% of graduates pursue a residency in their preferred specialty as indicated at the time of survey completion. There may be inherent inaccuracies in the self-reported debt and parental income for individuals entering these values into their questionnaire. While exact values are likely available to all graduates through borrower websites or tax forms, we cannot exclude the possibility that some or many used estimations or approximations to determine these figures. However, we have no reason to believe that these potential estimations could bias the results in a particular direction. Our data are limited to that available within the AAMC graduation and matriculation questionnaires. Other variables that may be of interest in the association of educational debt were outside the scope of this study (e.g., financial attitudes/behaviors, history of default).

Our study adds the following new insights. US medical graduates entering neurology with any educational debt decreased by 11% from 2010 to 2021. However, mean debt balances among indebted graduates also increased by $29,000, leading to the concentration of debt among fewer graduates who tended to be from lower-income families and more often identified with URiM groups. Furthermore, subspecialties that often manage patients in the outpatient setting, with limited inpatient and procedural care, may have greater debt with respect to their compensation. More work is needed to identify policies and efforts that will improve equitable access to medical education with an emphasis on workforce diversity and ensure the alignment of physician incentives and societal goals. Finally, nearly half of 2021 neurology-bound graduates plan to use tax-payer funds for loan forgiveness. As the PSLF program ages, the extent to which this loan forgiveness mechanism successfully relieves debt for medical graduates needs further investigation and critique regarding the equity of program benefits.

## Appendix Authors

**Table:**

Name	Location	Contribution
Shubhdeep V. Ahden, DO	University of Rochester Medical Center, NY	Drafting/revision of the manuscript for content, including medical writing for content; study concept or design; analysis or interpretation of data
Wasim Zatar, MBA	University of Rochester Medical Center, NY	Drafting/revision of the manuscript for content, including medical writing for content; analysis or interpretation of data
David N. Herrmann, MBBCh	University of Rochester Medical Center, NY	Drafting/revision of the manuscript for content, including medical writing for content; analysis or interpretation of data
E. Ray Dorsey, MD, MBA	University of Rochester Medical Center, NY	Drafting/revision of the manuscript for content, including medical writing for content; analysis or interpretation of data
Benjamin P. George, MD, MPH	University of Rochester Medical Center, NY	Drafting/revision of the manuscript for content, including medical writing for content; major role in the acquisition of data; study concept or design; analysis or interpretation of data

## Glossary

AAMC: Association of American Medical Colleges
E/M: evaluation and management
GQ: Graduation Questionnaire
IQR: interquartile range
LCME: Liaison Committee on Medical Education
MSQ: Matriculating Student Questionnaire
PSLF: Public Service Loan Forgiveness
URiM: underrepresented in medicine
